# A quantitative analysis of qualitative studies in clinical journals for the 2000 publishing year

**DOI:** 10.1186/1472-6947-4-11

**Published:** 2004-07-22

**Authors:** Kathleen Ann McKibbon, Cynthia S Gadd

**Affiliations:** 1 Center for Biomedical Informatics 8084 Forbes Tower, 200 Lothrop Street, University of Pittsburgh, Pittsburgh, PA USA 15213-2582; 2 Health Information Research Unit Department of Clinical Epidemiology and Biostatistics, McMaster University Faculty of Health Sciences Hamilton, Ontario Canada L8S 1V8

## Abstract

**Background:**

Quantitative studies are becoming more recognized as important to understanding health care with all of its richness and complexities. The purpose of this descriptive survey was to provide a quantitative evaluation of the qualitative studies published in 170 core clinical journals for 2000.

**Methods:**

All identified studies that used qualitative methods were reviewed to ascertain which clinical journals publish qualitative studies and to extract research methods, content (persons and health care issues studied), and whether mixed methods (quantitative and qualitative methods) were used.

**Results:**

60 330 articles were reviewed. 355 reports of original qualitative studies and 12 systematic review articles were identified in 48 journals. Most of the journals were in the discipline of nursing. Only 4 of the most highly cited health care journals, based on ISI Science Citation Index (SCI) Impact Factors, published qualitative studies. 37 of the 355 original reports used both qualitative and quantitative (mixed) methods. Patients and non-health care settings were the most common groups of people studied. Diseases and conditions were cancer, mental health, pregnancy and childbirth, and cerebrovascular disease with many other diseases and conditions represented. Phenomenology and grounded theory were commonly used; substantial ethnography was also present. No substantial differences were noted for content or methods when articles published in all disciplines were compared with articles published in nursing titles or when studies with mixed methods were compared with studies that included only qualitative methods.

**Conclusions:**

The clinical literature includes many qualitative studies although they are often published in nursing journals or journals with low SCI Impact Factor journals. Many qualitative studies incorporate both qualitative and quantitative methods.

## Background

Quantitative studies provide answers or insights for many important questions or issues in health care and clinical research. Other important questions dealing with why, how, contexts, and experiences of individuals or groups, can be best addressed using qualitative methods. Other issues benefit from interleaving or integration of both research traditions. Miller and Crabtree [1], describe their experiences working in family medicine, a clinical domain where balancing qualitative and quantitative research styles benefits both patients and their families and health care professionals. They embrace holding "quantitative objectivism in one hand and qualitative revelations in another" and encourage others to use findings from both paradigms in understanding and practicing effective health care. Creswell and colleagues expand on this theme by stating that "When used in combination, both quantitative and qualitative data yield a more complete analysis, and they complement each other" [2].

Most studies in the major clinical journals have been quantitative studies. Very few qualitative studies and even fewer that combine both qualitative and quantitative approaches are published. An example of the breadth of qualitative studies and how findings and results can be combined across paradigms is a study by Jolly and Wiles [3,4] who used mixed methods to study a nurse-led intervention for 422 adults after myocardial infarction and 175 adults with new-onset angina in 67 general practices in the United Kingdom. Their study showed statistically insignificant results at 1 month for eating healthy food, participating in exercise programs, and successful smoking cessation. Although patients in the nurse-led group were more likely to attend a rehabilitation program (37% vs. 22%, P = 0.001) attendance was disappointingly low. The researchers interviewed a group of patients using qualitative methods and found that people felt survival after a myocardial infarction indicated that the event had not been all that serious. Health care professionals often communicated simplified data about recurrence and being "back to normal" in 6 weeks. Because of these two issues, patients felt that their cardiac problems had probably been mild and therefore were not sufficiently motivated to implement major lifestyle changes.

Another example of the use of mixed methods was research done by Willms and Wilson and their colleagues [5-7] on smoking cessation. They found the meanings that patients who smoked attributed to their cigarettes (peer acceptance, coping during a time of stress and feeling out of control, feeling more like an adult, and smoking as more glamorous, tough, and rebellious) had more influence on cessation than did such external conditions as nicotine gum or counseling. Until the complex issues of why individuals smoke were dealt with, few were motivated to change their attitudes towards smoking and thus stop smoking.

Another effective example of integrated qualitative (ethnography) and quantitative (epidemiology) methods was a study done by Borkan and colleagues [8] to determine predictors of recovery after hip fracture in elderly patients. Traditional predictors such as age, type of break, and comorbidity, were collected by using standard questionnaires. In-depth interviews were used to collect injury narratives focusing on internal explanations of the fracture, sense of disability, and view of the future after hip fracture. None of the epidemiology factors predicted successful outcomes but those who perceived their fracture as more external or mechanical as opposed to an internal or organic problem (e.g., related to chronic disease) were more likely to have good recovery. Persons who perceived their disability in the context of autonomy, independence, and connection with the outside world also showed better ambulation at 3 and 6 months than persons with a more narrow and confined view of the fracture and its resulting disability.

Donovan and colleagues [9] used mixed methods to study prostate cancer screening and treatment choices to determine why study recruitment was lower than expected. Rousseau and Eccles and their colleagues [10,11] used qualitative methods (case interviews) to explain the limited use of computerized guidelines for asthma and angina in a primary care study done in the United Kingdom. Many other examples exist; Creswell and colleagues describe 5 additional mixed methods studies in primary care as well as provide criteria for evaluating mixed methods studies [2].

We postulate that qualitative studies, either stand-alone reports or studies with mixed methods, are occurring more frequently in health care. This paper was done to describe the publishing of qualitative studies in 1 year of clinical literature, document and present the range of content and techniques in these studies, and establish a baseline for subsequent studies. We defined our sample to include all articles published in a set of major general medical, mental health, or nursing journals during 2000. We determined how many qualitative studies were published and in which journals, and extracted design methods and healthcare content, and how often studies used mixed methods and analyses. Because the nursing literature published a higher proportion of qualitative studies in our sample we also compared studies published in nursing journals with other journals to ascertain if quantitative differences exist across disciplines in the use of qualitative methods. Our analysis is a quantitative review of qualitative studies in health care in 2000.

## Methods

The Health Information Research Unit of the Department of Clinical Epidemiology and Biostatistics, Faculty of Health Sciences at McMaster University in Hamilton, Ontario, Canada was the editorial office for four evidence-based summary journals in 2000: *ACP Journal Club *(internal medicine content), *Evidence-Based Medicine *(family/general practice content), *Evidence-Based Nursing *(general care nursing content), and *Evidence-Based Mental Health *(mental health care content). Their purpose is to provide enhanced abstracts and commentaries on important high-quality original studies and review articles for their respective clinical audiences. To identify these studies and review articles, 6 research staff read major clinical journals to ascertain if articles were in 1 or more categories of therapy, diagnosis, prognosis, etiology, economics, clinical prediction guides, differential diagnosis, and qualitative studies and if so, did each meet predefined methodology criteria for study quality[12]. For 2000 we intensified our data collection to provide data to update and develop new clinical retrieval searching hedges for MEDLINE, PsycINFO, CINAHL, and EMBASE using methods described by Haynes and colleagues[13]. One hundred and seventy journals provided data for this article.

The staff of the Health Information Research Unit has established quality criteria for the 8 categories of clinical literature that must be met before articles are judged appropriate for clinical application and publication in an abstract journal. Qualitative studies have 3 criteria:

• content relates to how people feel or experience certain situations, specifically those that relate to health care

• data collection methods and analyses are appropriate (primary analytical mode is inductive rather than deductive)

• units of collection and analysis are ideas, thoughts, concepts, phrases, incidents, or stories that become categories or themes.

The reading methods have been developed during the past 13 years and inter-rater reliability kappa (chance adjusted agreement) for identifying categories and applying criteria is consistently > 80%.

For this paper, KAM, one of the readers, analyzed the qualitative studies. Qualitative systematic reviews were excluded leaving only reports of original studies. These were assessed to extract journal title, qualitative study type, data collection methods, research question, persons studied, setting, and disease or health condition considered. In addition, studies with mixed methods were further analyzed although we did not use stringent criteria for assessing the quality [2] of the combination of methods. We identified mixed methods articles using a loose criterion of "some numerical or statistical analysis of quantitative data or qualitative data that had been turned into quantitative data". (An example of quantifying qualitative data is the study done by Borkan and colleagues [8] on hip fracture.) The analysis had to be fairly substantial–for example, a simple descriptive analysis of baseline demographics of the participants was not sufficient to be included as a mixed methods article.

In addition, Giacomini and Cook [14,15], as part of the Evidence-Based Working Group in the Users' Guides to the Medical Literature, describe attributes that they have identified as belonging to high-quality qualitative studies: participant selection, data collection, and analysis methods. These aspects were also extracted for analysis in this report.

Data were taken from article abstracts and if needed, the full text was reviewed. Methodologies assessed were phenomenology, grounded theory, ethnography, case studies, narrative analysis, participant action, critical incident techniques, and discourse analysis. Author descriptions were used and if an additional methodology was found it was added to the list of types using definitions and descriptions from the Handbook of Qualitative Analysis, 2^nd ^edition by Denzin and Lincoln [16]. Data collection and sampling procedures were also extracted. Multiple designations were allowed. To assess the reproducibility a random 10% (n = 35) sample of citations was reviewed using predefined decision rules by another researcher trained in research methods.

## Results

The 170 journals included 60 330 articles of which 31 496 (52%) contained original data or were review articles. 3830 of these (6%) passed criteria for being high-quality and clinically relevant in 1 of the 8 categories. 367 articles met quality criteria for original studies or reviews of qualitative studies. Table 1 lists the journals that published at least 1 qualitative study. Twelve systematic reviews were excluded leaving 355 qualitative studies for assessment. Approximately 0.6% of all articles in the 170 journals and 9% of all high-quality, clinically relevant studies were qualitative studies.

The reproducibility of the categorization was measured by kappa (chance adjusted agreement): 0.92 for disease/condition, 0.83 for groups studied, 0.81 for setting, 0.73 for data collection, and 0.63 for data analysis type. The agreement for data analysis type was disappointing but not surprising in that 20% of the studies did not label their analyses necessitating assignment of analysis type by the data extractors. Agreement was low for participant selection methods (kappa 0.5) and therefore data on participant selection methods are not reported.

The 355 qualitative studies appeared in 48 journals (mean 7.4 articles per journal, range 1 to 86). These 48 journals were only 28% of the 170 clinical journals being read. Most of the qualitative studies were published in nursing journals: The 17 nursing titles included 214 qualitative studies (61% of all of the qualitative studies).

Few qualitative studies were published in the high-circulation, general healthcare journals. Using SCI Impact Factor ranking for 2000, only 4 of the top 20 journals (Table 2) published qualitative studies. These 4 journals published 15 qualitative studies with *BMJ *publishing 12. The highest-ranking journal with qualitative studies was *Annals of Internal Medicine*, ranked number 6. *JAMA*, ranked number 2, published articles about qualitative studies in 2000 [14,15] but did not publish any qualitative studies.

### Mixed qualitative and quantitative studies

37 qualitative studies (11%) included qualitative and quantitative methods and analyses. These were published in 17 journals with only 1 article in *BMJ *from the top 20 journal titles in Table 1. *Social Science and Medicine *published 10 of these mixed methodology studies–the most of any title studied.

### Content

Content of the studies is shown in Table 3. Many studies dealt with a range of participants and settings. Patients (56%), family (22%), and other non-health care professionals (14%) were studied more often than health professionals (nurses (21%), physicians (11%), and others (5%)). Non health care settings occurred more often with home or similar settings being studied in 44% of studies and other community settings in 16%. Health care settings were the hospital (25%), clinic (17%), nursing home (5%), and the emergency department (2%).

Disease/condition breakdowns represented common health care situations: cancer (11%), mental health (10%), pregnancy and childbirth (9%), cerebrovascular disease (10%), general issues such as vaccinations or Internet use, and nonspecific spectrum of diseases (e.g., all patients in a clinic) (12%). Many uncommon issues were also assessed. For example, Tongprateep [17] reports a phenomenology study designed to help nurses better understand essential elements of spirituality and health among rural Thai elders.

Analysis of the 37 articles with mixed methods showed similar patterns for settings, persons studied, and disease/condition evaluated except that more physicians were studied (P < 0.025) and more situations dealing with injury (P < 0.001) were evaluated. For the 211 articles in Nursing journals, very little difference was also seen except that fewer physicians were studied (P < 0.001) and more studies were done outside clinical settings (P < 0.001).

Phenomenology (37%), grounded theory (35%), and ethnography (18%) were used most often with some case studies (7%), narrative analysis (6%), participant action (3%) research, critical incident techniques (1%), and discourse analysis (1%) (Table 4). More than one qualitative method was used in 8% of studies. This pattern of methodology choice was similar for the 37 mixed methods studies and the 211 Nursing articles except that mixed studies methods used relatively more case studies and the Nursing studies used fewer of them (P < 0.025). The mixed methods studies did not included participatory action research, critical incident technique, or discourse analysis studies, methods that could be difficult to combine with quantitative studies.

Semi-structured interviews were used (77%) with some focus groups (18%) and observation (14%). These methods are major data gathering techniques in qualitative studies. Questionnaires (7%), document analysis (6%), and structured (4%) and unstructured interviews (1%) were used less often. For mixed methods studies, patterns were similar although questionnaires were used more frequently (24% vs. 7%, P < 0.01). Nursing studies did not differ for data gathering techniques.

Sampling is important in all studies–often no single right way exists for a study question. Purposive, snowball, and theoretical sampling are often used in qualitative studies and random and consecutive sampling for quantitative studies. All methods were represented in this analysis but the breakdowns are not reported because of low inter-rater agreements for categorization and missing author information.

## Discussion

In 2000 the major clinical journals published many qualitative studies–approximately 9% of all high-quality, clinically relevant articles. Most of the qualitative studies were reports of original research although 12 (3%) were systematic reviews. Most of the qualitative studies were in nursing journals although some medical journals such as *BMJ *and *Annals of Internal Medicine *also published several. Three of the high circulation medical journals (*New England Journal of Medicine, Lancet, *and *JAMA*) and 16 of the top 20 clinical journals, based on SCI Impact Factors, did not publish any qualitative studies. This is likely a reflection on the emphasis on a positivist, numerical approach that many of these journals embrace. The difference in proportion of qualitative studiers published in nursing journals is probably because of two historical, but linked factors. Qualitative studies have roots in women's studies and the nursing profession has always dealt with the patient as much more of a whole person rather than basic sciences facts and numbers. Both of these factors lead to more emphasis on understanding and embracing qualitative methods for research and practice. This view is substantiated by the fact that MEDLINE indexes most of the qualitative studies under the term Nursing Methodology Research until 2003.

A substantial proportion of the qualitative studies (11%) included both qualitative and quantitative (mixed) data. In general, these mixed methods studies were similar to the single methodology studies except they did more assessments of physicians and relied more on questionnaires to gather data for analysis. The presence of these mixed methods or multipardigmatic studies as described by Miller and Crabtree [1] and Creswell [18] is encouraging for those who espouse harnessing methodologies appropriate for exploring, explaining, and interpreting the complexities and ranges of issues in health care practice and research.

It is also interesting comparing qualitative studies in Nursing and non-Nursing journals. Regardless of the differences in proportion of qualitative studies published, from a content point of view few differences exist between the Nursing and non-Nursing journals except that more physicians were studied in the non-Nursing journals and fewer studies were done in clinical settings–not unsurprising findings. This indicates that the content and methods of qualitative studies seem to be similar across disciplines or if the methods are combined with quantitative methods.

This review of the publication of qualitative studies is limited in several ways. The proportion of journals studied was very low in relation to the total number of journals published. MEDLINE indexes over 4000 journals and this number is still a relatively small proportion of all journals that deal with health care. In addition, all of the journals searched were published in English so we do not know about qualitative studies in other languages. Although our criteria were relatively strict for including qualitative studies, our criteria for mixed methods studies could certainly have been stronger. We did not count the number of high-quality quantitative studies that could have included some qualitative analyses. We studied only 1 year of publishing; much could have changed since 2002.

Qualitative studies provide insight into social, emotional, and experiential aspects of health and health care and as such, they have an important place in understanding health and health care. Hopefully more studies will be published and more will be published in the high impact (high circulation) journals. This paper provides a basis for measuring increases.

## Conclusion

Qualitative studies are being done and are published in a wide range of healthcare journals. These journals however are not the highest impact journals. It is encouraging to see that the number of qualitative studies that were published in 2000 and also the number of studies that combined qualitative and quantitative methods. More can be done however to complete and publish qualitative studies, and where appropriate, integrate the best of both methodologies. Both qualitative and quantitative researchers and clinicians need to work together to make this happen. Journal editors can also encourage submission of qualitative and mixed methods studies and facilitate publication of those they do receive.

## List of Abbreviations

SCI ISI Science Citation Index

## Competing interests

None declared.

## Author Contributions

This work was done in partial fulfillment of PhD requirements for KAM. Both authors have supplied intellectual input in designing and implanting the survey. KAM has collected and analyzed the data and both authors have contributed to writing the paper and agree on its content.

## Pre-publication history

The pre-publication history for this paper can be accessed here:



## Figures and Tables

**Table 1:** Journal Title with number of Original and Review Qualitative Studies for 2000

**Journal Title**	**Number of Qualitative Studies**
Journal of Advanced Nursing	88
**Social Science and Medicine**	**34**
Qualitative Health Research	30
**Journal of Clinical Nursing**	**22**
Western Journal of Nursing Research	15
**Cancer Nursing**	**14**
BMJ	12
**Research in Nursing and Health**	**12**
Midwifery	10
**Public Health Nursing**	**10**
Family Practice	9
**ANS Advances in Nursing Science**	**8**
British Journal of General Practice	8
**Heart and Lung**	**8**
Journal of Nursing Scholarship	8
**Patient Education and Counseling**	**8**
Journal of Pediatric Nursing	6
**Clinical Nursing Research**	**5**
Journal of Family Practice	5
**Journal of General Internal Medicine**	**5**
Birth	4
**Health Education and Behavior**	**4**
Archives of Pediatrics and Adolescent Medicine	3
**Canadian Journal of Nursing Research**	**3**
Canadian Journal of Public Health	3
**Journal of Pediatric and Oncology Nursing**	**3**
Neonatology Review	3
**Pediatrics**	**3**
Qualitative Health Care	3
**Applied Nursing Research**	**2**
Psychiatric Services	2
**Addiction**	**1**
American Journal of Psychiatry	1
**American Journal of Public Health**	**1**
Annals of Emergency Medicine	1
**Annals of Internal Medicine**	**1**
Archives of Physical Medicine and Rehabilitation	1
**Australian and New Zealand Journal of Psychiatry**	**1**
BJOG (British Journal of Obstetrics and Gynaecology)	1
**British Journal of Psychiatry**	**1**
CMAJ (Canadian Medical Association Journal)	1
**Diabetes Care**	**1**
Fertility and Sterility	1
**International Journal of Geriatric Psychiatry**	**1**
Journal of Epidemiology and Community Health	1
**Journal of Psychosomatic Medicine**	**1**
Nursing Research	1
**Western Journal of Medicine**	**1**

**Table 2:** Top Health Care Journals with SCI Impact Factor for 2000 and Number of Qualitative Studies

**Journal Title**	**SCI Impact Factor**	**Qualitative Studies**
New England Journal of Medicine	29.521	0
**JAMA**	**15.402**	**0**
Archives of General Psychiatry	11.778	0
**Circulation**	**10.893**	**0**
Lancet	10.232	0
**Annals of Internal Medicine**	**9.833**	**1**
Annals of Neurology	8.480	0
**Journal of the American College of Cardiology**	**7.082**	**0**
Psychological Bulletin	6.913	0
**Arthritis and Rheumatism**	**6.841**	**0**
American Journal of Psychiatry	6.577	1
**Archives of Internal Medicine**	**6.055**	**0**
American Journal of Medicine	5.960	0
**American Journal of Respiratory and Critical Care Medicine**	**5.443**	**0**
Gut	5.386	0
**BMJ**	**5.331**	**12**
Hypertension	5.311	0
**Diabetes Care**	**4.992**	**1**
Journal of Infectious Diseases	4.988	0

**Table 3:** Content for Qualitative Studies (n = 355), Mixed Methodology Studies (n = 37), and Studies from Nursing Journals (n = 211)

**Category**	**All studies**	**% All studies**	**# Mixed Methods**	**% Mixed Methods**	**# Nursing Journals**	**% Nursing Journals**
**Groups studied**						
Patients	198	56	17	45	114	54.0
**Family**	**78**	**22**	**9**	**24**	**52**	**24.6**
Nurses	73	21	6	16	55	26.1
**Other People**	**49**	**14**	**0**	**0**	**48**	**22.7**
Physicians	38	11	8**	21	9*	4.3*
**Other Health Care Professionals**	**20**	**6**	**1**	**3**	**12**	**5.7**
Settings						
Home or non institution	155	44	16	42	100	47.4
**Hospital**	**87**	**25**	**10**	**26**	**65**	**30.1**
Clinic	62	17	7	18	16*	7.6*
**Community**	**48**	**14**	**7**	**18**	**36**	**17.1**
Nursing Home	19	5	2	5	13	6.2
**Emergency Dept**	**6**	**2**	**1**	**3**	**2**	**0.9**
Church, Jail, Other	5	1	2	5	2	0.9
**Disease/Condition**						
Various	38	11	3	8	19	9.0
**Cancer**	**40**	**11**	**5**	**13**	**28**	**13.2**
Mental Health	38	10	3	8	26	12.3
**Pregnancy/birth**	**33**	**9**	**3**	**8**	**16**	**7.6**
Cerebrovascular Disease	34	10	2	5	22	10.4
**General Health**	**33**	**9**	**2**	**5**	**17**	**8.1**
Frail Elderly	29	8	3	8	21	10.0
**HIV/AIDS**	**21**	**6**	**3**	**8**	**11**	**5.2**
Drugs/Sex Trade	13	4	2	5	7	3.3
**Death and Dying**	**13**	**3**	**0**	**0**	**7**	**3.3**
Diabetes	9	3	2	5	4	1.9
**Critical Care**	**8**	**2**	**1**	**3**	**8**	**3.8**
Injury	7	2	5*	13	5	2.4
**Asthma**	**6**	**2**	**1**	**3**	**0**	**0**
Pain	7	2	1	3	3	1.4
**Smoking**	**4**	**1**	**1**	**3**	**2**	**0.9**
Miscellaneous disease/conditions	41	12	5	13	26	12.3

*P < 0.001 **P < 0.025. Note that totals do not add to 100% because studies often included several methods, settings, diseases, or populations.

**Table 4:** Methodologic Analysis of the Qualitative Studies (n = 355), Mixed Methods (n = 37), and Nursing Journals (n = 211)

**Category**	**# All studies**	**% All studies**	**# Mixed Methods**	**% for Mixed Methods**	**# Nursing Journals**	**% Nursing Journals**
Analysis Type						
Phenomenology	131	37	14	37	87	41.2
**Grounded Theory**	**124**	**35**	**10**	**26**	**74**	**35.1**
Ethnography	64	18	8	21	33	15.6
**Case Studies**	**24**	**7**	**5**	13*	**9**	4.3**
Narratives	21	6	2	5	12	5.7
**Participatory**	**13**	**4**	**0**	**0**	**10**	**4.7**
Critical Incident Technique	4	1	0	0	3	1.4
**Discourse Analysis**	**4**	**1**	**0**	**0**	**3**	**1.4**
Data Collection						
Semi-structured Interviews	272	77	27	71	167	79.1
**Observation**	**64**	**18**	**6**	**16**	**37**	**17.5**
Focus Groups	50	14	5	13	31	14.7
**Questionnaires**	**25**	**7**	9***	**24**	**14**	**6.6**
Documents	23	6	2	5	11	5.2
**Unstructured Interviews**	**13**	**4**	**1**	**3**	**10**	**4.7**
Structured Interviews	2	1	0	0	2	0.9

* P < 0.001 **P < 0.025 *** P < 0.01
